# Factors influencing maternal healthcare seeking in a highland region of Madagascar: a mixed methods analysis

**DOI:** 10.1186/s12884-021-03930-2

**Published:** 2021-06-16

**Authors:** Voahirana Tantely Andrianantoandro, Dolorès Pourette, Olivier Rakotomalala, Henintsoa Joyce Valentina Ramaroson, Rila Ratovoson, Feno M. Jacob Rakotoarimanana

**Affiliations:** 1 Unité Mixte Internationale-Résiliences (Université Catholique de Madagascar, Institut de Recherche pour le Développement), Université Catholique de Madagascar, BP 6059, 101 Antananarivo, Madagascar; 2 IRD, Ceped (IRD, Université Paris Descartes, INSERM), équipe SAGESUD, Paris, France; 3 Centre de recherche pour le développement (Université Catholique de Madagascar), Antananarivo, Madagascar; 4 Cellule Santé & Sciences Sociales (SaSS), Institut Pasteur de Madagascar and Université de Montréal, Antananarivo, Madagascar; 5 Unité d’Epidémiologie et de Recherche Clinique, Institut Pasteur, Antananarivo, Madagascar; 6 USAID ACCESS - Management Sciences for Health, Institut Pasteur, Antananarivo, Madagascar

**Keywords:** Maternal healthcare seeking, Determinants, Highlands of Madagascar, Mixed methods

## Abstract

**Background:**

In Madagascar, maternal mortality remains stable and high (426 deaths per 100,000 live births). This situation is mainly due to a delay or lack of use of maternal healthcare services. Problems related to maternal healthcare services are well documented in Madagascar, but little information related to maternal healthcare seeking is known. Thus, this paper aims to identify and analyze the factors that influence the utilization of maternal services, specifically, the use of antenatal care (ANC) during pregnancy and the use of skilled birth attendants (SBAs) at delivery.

**Method:**

We used quantitative and qualitative approaches in the study. Two communes of the Vakinankaratra region, which are located in the highlands, were the settings. Data collection occurred from October 2016 to July 2017. A total of 245 pregnant women were included and followed up in the quantitative survey, and among them, 35 participated in in-depth interviews(IDIs). Logistic regressions were applied to explore the influencing factors of antenatal and delivery healthcare seeking practices through thematic qualitative analysis.

**Results:**

Among the 245 women surveyed, 13.9% did not attend any ANC visits. School level, occupation and gravidity positively influenced the likelihood of attending one or more ANC visits. The additional use of traditional caregivers remained predominant and was perceived as potentially complementary to medical care. Nine in ten (91%) women expressed a preference for delivery at healthcare facilities (HFs), but 61% of births were assisted by a skilled birth attendant (SBA).The school level; the frequency of ANCs; the origin region; and the preference between modern or traditional care influenced the use of SBAs at delivery. A lack of preparation (financial and logistics problems) and women’s low involvement in decision making at delivery were the main barriers to giving birth at HFs.

**Conclusion:**

The use of maternal healthcare services is starting to gain ground, although many women and their relatives still use traditional caregivers at the same time. Relatives play a crucial role in maternal healthcare seeking. It would be necessary to target women’s relatives for awareness-raising messages about ANC and childbirth in healthcare facilities and to support and formalize collaborations between traditional healers and biomedical caregivers.

## Background

Sustainable development goal (SDG) 3.1 aims to reduce maternal mortality to under 70 deaths per 100,000 live births by 2030. According to the World Health Organization (WHO), in 2017, maternal mortality was 211 deaths per 100,000 live births at the world level, and 86% of all deaths occurred in the sub-Saharan African region, where the ratio was 542 deaths per 100,000 live births [1]. In Madagascar, maternal mortality remains stable and high. From 1997 to 2010, maternal mortality decreased from 498 to 478 deaths per 100,000 live births [2, 3]. In 2018, the rate was 426 deaths per 100,000 live births [4], which shows slight progress; still, a major effort is needed to achieve the SDG for maternal health.

According to previous studies and surveys, the main cause of maternal deaths in Madagascar is the delay or absence of medical care in cases of obstetric complications [4, 5]. In 2018, the Multiple Indicator Cluster Survey (MICS) indicated that only 30.3% of pregnant women received four antenatal follow-ups as recommended by the WHO and that only 49% of births were assisted by a skilled birth attendant (SBA) [4]. The majority of deliveries took place at home and were assisted by a *reninjaza*, a traditional birth attendant (TBA). A *reninjaza* does not receive any medical training but provides care and advice to women during pregnancy, delivery and the postnatal period [6, 7]. However, their roles vis-à-vis the healthcare system are controversial. In fact, the Ministry of Health (MH), in line with WHO recommendations, promotes maternal and child healthcare with skilled attendants to prevent, detect and treat any complications throughout pregnancy and delivery [8].

Access to healthcare remains difficult in Madagascar. The country is one of the poorest countries in the sub-Saharan African region, with a poverty rate of 70.5% in 2019 [9]. In 2018, only 8% of Malagasy people had health insurance. The majority of primary healthcare facilities (PHFs) are not readily accessible. In fact, 25.8% of Malagasy people live more than 10 km from HFs; the majority are understaffed, and 53.5% are not accessible year-round [5, 10]. In 2018, 51% of PHFs had only one caregiver, 50.3% did not have a doctor, and only 17.7% were available to receive basic emergency obstetric care [5]. Moreover, a lack of equipment and stockouts of medicine and supplies are constant challenges [11–13].

According to the Service Availability and Readiness Assessment (SARA) survey performed by John Snow, Inc. (JSI) and the US Agency for International Development (USAID), in 2018 in 7 regions, only 51% of PHFs required the minimum package of consumables for maternal and child health activities (such as nutritional supplements, a tetanus vaccine, insecticide-treated nets (ITNs), and medications for intermittent preventive treatment). Concerning obstetric and neonatal care, every PHF possessed basic equipment for delivery (beds, etc.), but only 47% of them had essential medications for mothers [14].

This deficiency of the public health system leads the population to seek care other than biomedical care. This alternative care includes home care, self-medication and traditional care provided by traditional healers [6, 11, 15, 16]. These recourses are not mutually exclusive: people simultaneously use biomedical systems, home care, and traditional healers depending on their financial means, the possibilities available to them in their vicinity, and the evolution of their health status.

In Africa, the following factors have often been identified as determinants of healthcare seeking among pregnant women: the formal school level (both of the husband/partner and the woman); religion; age; ethnicity; the level of women’s autonomy; socioeconomic status; pregnancy experience; knowledge of pregnancy danger signs; decision making on the place of delivery; place of residence; distance from the residence to an HF; the cost or gratuity of maternal healthcare; and contextual factors [17–29]. In Madagascar, delays in seeking care are due to a lack of identification of need, economic barriers and geographical distance [4, 13]. People usually first attempt self-medication or traditional care that is associated with common cultural practices (massages, herb taking, etc.), which could have negative impacts on maternal and child health [11]. Thus, in view of the high maternal mortality rate, access to maternal and child healthcare services remains a challenge for the Malagasy government. Apart from supply-side issues, it is therefore necessary to understand the real issues in pregnant women’s behavior to implement effective interventions and to better achieve maternal health goals.

The main objective of this work is to identify and analyze the determinants of the use of antenatal care (ANC) during pregnancy and the use of SBA services during delivery. The analysis focused on describing maternal healthcare practices and identifying their associations with the obstetric history, socioeconomic and cultural factors, and individual logic related to these practices.

## Methods

A mixed methods approach (both quantitative and qualitative) was adopted to collect data from October 2016 to July 2017. For the quantitative survey, we conducted a cross-sectional study with two measures. The first data collection phase was carried out from October–November 2016 with pregnant women in the second trimester of pregnancy. A follow-up was performed from June-July 2017 with the same women approximately 3 months after they had given birth. For the qualitative approach, in-depth interviews were performed with a sample of healthcare providers, including matrons, pregnant women/mothers, parents of children under 5 years of age and community health workers (CHWs). The quantitative and qualitative approaches were complementary, and both were conducted at the same study site to allow the triangulation of information.

### Study setting

Vakinankaratra is located in the southern highland region 160 km from the capital of Antananarivo. We chose this region because it is one of the six regions in the country with the lowest levels of maternal mortality (< 95 deaths per 100,000 live births, which is the average rate from 2010–2017 as reported by the MH) [30]. The region is divided into 7 districts and has a population of approximately 2,074,358 people (or 8.1% of the Malagasy population). Antsirabe is the capital of the region, which is located 160 km from the national capital of Antananarivo. The ratio of formal HF use is 1/11,034 people, which is lower than the national ratio (1/8, 181 people in 2018) [31]. The formal HFs that offer maternal services consist of two public hospitals for women requiring cesareans, 157 public HFs and 14 private clinics. In 60% of communes (the commune is an intermediate administrative subdivision of the Malagasy political system), the closest HF is located within a 3-h walking distance of a residence [32].

### Quantitative approach

#### Sampling

All pregnant women at the community level in the second and third trimesters of pregnancy who were residents of the study zone for over 6 months at the time of the first phase of data collection were eligible for the study. A one-stage cluster sampling method stratified by rural and urban areas was performed on select communes. The urban commune of the Mahazoarivo Avarabohitra District of Antsirabe I and the rural commune of Andranomanelatra, Antsirabe II were selected. The administrative boundary database of the Ministry of the Interior served as the sample frame. All fokontany (a fokontany is the smallest administrative boundary unit in Madagascar) within selected communes were included. In the absence of an official pregnant women database at any level, selection was based on the Bacterial infections and antibiotic resistant deases research study (BIRDY) [33] and field experiences, which were conducted in the Antananarivo (urban) and Moramanga (rural) communes from 2012 to 2017. If the study team could identify only approximately 3 pregnant women in the third trimester per month within a fokontany, then by considering the 2nd trimester among the study inclusion criteria, we estimated that we could find approximately 5 eligible women per fokontany per month in the overall 31 fokontany of selected communes. Thus, the field survey was enlarged to 45 days, and accordingly, an exhaustive sampling of women was ensured.

For the calculation of the population-based sample size, we used Epitools (Sergeant and ESG, 2016). The calculation was as follows: *n* = (Z2 x P (1/P))/C2.

In 2016, the number of expected births in Vakinankaratra was 87,916, and the percentage of deliveries at HFs was 25% [34]. Based not only on this percentage as the value for *p* but also on a 5% margin of error and a proportion of 20% of women lost to follow-up between the two surveys, a sample size of 244 was set.

#### Material

The determinants of maternal healthcare service utilization can be categorized along the four dimensions of the geographical location of healthcare facilities (HF), availability of care, cost of care and acceptability (preference between the modern system and the traditional system for healthcare) of maternal healthcare services [35–40]. We considered these four dimensions in our study and added information related to pregnant women [17–29]. The questionnaire for the first survey mostly included sections on demographics and socioeconomic characteristics, obstetric history, the use of maternal healthcare services and knowledge about ANC. The questionnaire used in the second survey was focused on delivery (circumstances and outcomes), immediate postnatal care and children’s general healthcare seeking. All questionnaires used were pretested prior to the first field survey.

### Statistical analyses

In Madagascar, until December 2020, the national health management information system (NHMIS) tracked only the number of women who had the first and fourth antenatal care visits despite the new WHO recommendation published in 2016 [41]. In fact, regarding implementation strategies, the MH has adopted a minimum of 8 contacts with medical staff or CHWs but still recommends at least 4 formal ANCs with qualified medical staff [42]. Therefore, for the statistical analyses, we focused on ANC practice rather than all contacts according to the MH strategies, and only ANCs were well documented at the time of data collection. The outcome variable was then categorized into the following 3 groups: a) zero or no ANC done; b) 1 to 3 ANC visits; and c) 4 or more ANC visits. This categorization was realized to better explore the different levels of healthcare practice during the pregnancy period with their potential associated specific influencing factors.Therefore, multinomial logistic regressions were performed.

For the analyses of healthcare seeking at the time of delivery, practices were dichotomized into women who delivered with or without the assistance of skilled birth attendant staff according to the Demographic Health survey (DHS) tracked indicator [3, 5]. Thus, a binary logistic regression analysis was carried out to identify the factors that influence practice. The relative risk ratio (RRR), adjusted odds ratio (AOR) and their 95% confidence intervals (CIs) are reported according to the multivariate and binary logistic analyses. We considered a *p*-value of < 0.05 to be statistically significant in both models.

The following explanatory variables used in the multivariate analysis were identified by univariate analyses and/or were quoted in the literature: the distance to the healthcare center; sociodemographic variables (such as age and school level and marital status); income variables; obstetric history (number of previous pregnancies); and the preference between the biomedical system and the traditional system for healthcare seeking.

The analysis was performed with STATA v13.0 (StataCorp College version 2013).

### Qualitative approach

The qualitative data collection took place in October 2016 during the first survey. It was carried out by two investigators trained in qualitative data collection and ethnographic methods. The interview guides covered diverse topics areas, including maternal health knowledge and the health of children under 5 years of age, the perception and use of formal or traditional healthcare services and the specific healthcare practices related to pregnancy, delivery and postpartum care.

The qualitative research included IDIs and focus groups. We randomly selected 35 pregnant women from the sample to participate in IDIs and 19 parents of children under 5 years of age (18 mothers and 1 father) to participate in focus groups and IDIs. Furthermore, 2 grandmothers were recruited through snowball sampling, and they responded to an IDI. We also conducted IDIs with different stakeholders involved in maternal and child healthcare, including 7 community health workers (CHWs), 5 healthcare workers (2 doctors and 3 midwives), 10 *reninjaza* and 5 traditional healers.

For the pregnant women, the eligibility criteria were to have participated in the quantitative survey and to have given their consent to participate in the qualitative study. For the other respondents (parents of children under 5 years of age, community health workers, healthcare workers, and traditional caregivers), the eligibility criteria were to have lived in the study zone for at least 6 months at the time of the study.

All participants were informed of the objective of the study and signed an informed consent form. Each interview took place in the participant’s home in a quiet and isolated area and lasted approximately 45 min to 1 h. Each interview was conducted individually to avoid any possible influence of members of the respondent’s personal or professional entourage concerning his/her responses.

In-depth interviews were conducted by using semi-structured interview guides. We designed an interview guide for each category of respondent (7 interview guides in total). The interviews were conducted in Malagasy to better ensure the respondents’ understanding of the questions and provide more ease and confidence between the interviewer and the respondents. All interviews were recorded, fully transcribed and translated into French.

The transcribed interviews were subjected to two types of analysis: a biographical analysis and a cross-sectional thematic analysis. The biographical analysis consisted of reconstructing the life paths and, in particular, the reproductive paths or healthcare paths of each person interviewed. We carried out the cross-sectional thematic analysis by using a coding plan that we prepared on the basis of the themes and subthemes discussed during the interviews. Each theme and subtheme were analyzed across all the interviews to emphasize the salient features related to these themes and subthemes.

## Results

During the first data collection phase, 245 pregnant women residents of the two communes were included and surveyed. In the second survey, at which point all women were expected to have given birth, 14 women were lost to follow-up. Thus, the analysis relates only to 229 women who gave birth.

### Socioeconomic status and obstetric characteristics of the respondents

Table 1 shows that the survey participants were mostly native to the Vakinankaratra region. The mean age of the women was 24.5 years (± SD = 6.5), and one in ten of them was under 18 years old. The majority of the participants had attended school, but half of them stopped at the primary level, and only 3% had attended high school. Women in a couple were either legally married or in a common-law relationship. Concerning the participants’ economic situation, more than three-quarters of the women had income-generating activity, but for more than half of their households, the family income was under USD $1.90 per day.

**Table 1:** Sociodemographic and geographic characteristics of the respondents (*n* = 245)

**Variables**	**Categories**	**N**	**%**
Type of residence	Urban	111	45.3
	Rural	134	54.7
Distance to healthcare facility	< = 5 km	203	83.3
	> 5 km	42	16.7
Age (years)	< 18	22	9.0
	> = 18- < = 24	97	39.6
	> = 25–49	126	51.4
School level	None	14	5.7
	Primary	110	44.9
	Secondary and above	121	49.4
Marital status	In couple	233	95.1
	Single	12	4.9
Occupation	Yes	190	77.5
	No	55	22.5
Subscription to a mutual health	Yes	26	10.8
	No	219	89.2
Family income	1 < rev < = 1.90^a^ USD	140	57.2
	> 1.90 USD	105	42.8
Origin	Other regions	21	8.6
	Vakinankaratra	224	91.4

ªInternational poverty line, World Bank, 2015

Regarding childbirth experiences, one in four women was in her first pregnancy, and for multigravidas, the mean parity was 3.5; 18% of women who had previously given birth had faced an obstetric complication (Table 2) that involved stillbirth (in 1/3 of cases) or spontaneous abortion (in 1/5 cases).

**Table 2:** Health status of the respondents (*n* = 245)

**Characteristics**	**Categories**	**N**	**(%)**
**Gravidity**	Primigravida	61	24.9
	2 to 4 gravida	148	60.4
	> 4 gravida	36	14.7
**Medical background**	No	224	91.4
	Yes	21	8.6
**Current heath problem**	No	228	93.1
	Yes	17	6.9
**Problem during a previous childbirth**	**Multigravida**	***n* = 184**	**%**
	Yes	33	17.9
	No	151	80.1

The women interviewed after the qualitative survey were between 18 and 41 years old (two women did not know their age), and the average age was 25 years. Slightly more than half of the women had attended only primary school, 10 had attended secondary school, 3 had attended high school, and 3 had continued on to a higher educational level. The majority (22 women) worked in agriculture, 6 had no paid employment, and 4 were shopkeepers. All of the women were legally or traditionally married. Half of the women interviewed had one or two children. Seven women did not have children, five had three children, and the others had four or more children (over nine children).

### Antenatal care practices and other resources during pregnancy

Among the women, 13.9% had never attended any ANC, and most of them were in the second trimester. In the third trimester, 40% of the women attended four ANCs or more as recommended while it was only 9.3% during the 2^nd^ trimester (Table 3).

**Table 3:** Distribution of women according to age of pregnancy and frequency of ANC performed by SBAs (*n* = 245)

**ANC frequency**	**Second trimester**		**Third trimester**	
	**N**	**%**	**N**	**%**
0	29	24.6	5	3.9
1	32	27.1	14	11.0
2	33	28.0	25	19.7
3	13	11.0	32	25.2
4	9	7.6	32	25.2
4 and more	2	1.7	19	15.0
Total	118	100	127	100

Notably, 56.7% of the women preferred using exclusively biomedical healthcare services (Table 4), and there was no significant difference between type of residence (*p* = 0,79). For the rest, the use of traditional caregivers (especially matrons/reninjaza) remained common.

**Table 4:** Distribution of the women according to their preference of healthcare system and type of residence (*n* = 245)

**Preference**	**Residence type**		**Total**
	**Rural**	**Urban**	
	**n (%)**	**n (%)**	**n (%)**
Modern health system only	64 (57.7)	75 (56.0)	139 (56.7)
Other (traditional or both systems)	47 (42.3)	59 (44.0)	106 (43.3)

### Preventive care during pregnancy

All women participating in the qualitative study felt that preventive care during pregnancy was essential. This care can be divided into three categories, namely, formal ANC visits, care from the *reninjaza* (TBA), and the habits to adopt until delivery.

#### Antenatal care visits

The pregnant women mostly used ANC out of concern for their health and the health of their baby. All the pregnant women interviewed considered it to be important to have antenatal consultations during pregnancy. Two main motivations were mentioned. First, the women said that they obtain ANC to monitor their health and the health of their baby. The medical training of the healthcare workers and their equipment allows certain abnormalities to be identified and remedied. Second, receiving ANC allows for them to obtain a notebook with records of their completed ANC visits. Having this notebook simplifies the administration matters at the healthcare facility in case of complications during delivery. The women think that if they do not have this booklet that attests to their prenatal care, then care at a healthcare facility would be refused.

#### Recourse to the *reninjaza (matrons)*

The women visited the *reninjaza* for several reasons. Through her massages, a *reninjaza* can feel whether the fetus has an acceptable size or if it is too large and can advise women to give birth at a healthcare facility if needed. The massages also allow the *reninjaza* to determine whether the fetus is well positioned for delivery. In addition, the *reninjaza* can massage a woman’s body to reposition the fetus. Some women reported that healthcare workers are not able to identify this type of problem during prenatal visits. The *reninjaza’s* massages are also necessary to reduce fatigue and back pain during pregnancy to help women perform their daily activities (e.g., agricultural and domestic activities).

For the women, massage and ANC are both important to ensure the good development of a pregnancy. These practices did not appear to vary much according to the place of residence. Most women combined both biomedical and traditional healthcare services by trying to make the different types of healthcare seeking complementary (for example, women received massages from a reninjaza for their well-being because this service is not available at healthcare facilities). This healthcare seeking trend shows that the women did not adopt a single type of healthcare but chose and used the best options for them. The *reninjaza* and doctors who participated in an in-depth interview agreed that both types of healthcare providers have specific skills and that women’s expectations about the care that they provide (ANC and massages) differ. However, some women avoided massages for fear of reprisals from doctors who prohibited this practice during pregnancy or believed that the massages were dangerous because *reninjazas* have no academic training in this practice.

#### Other preventive care

Finally, the women adopted some habits during pregnancy. To avoid a complicated delivery, the women observed dietary restrictions (the fetus must be small) and protected themselves against any source of heat (sun and fire) to prevent “lafika” (when the placenta sticks to the uterus). Another resource for the women during pregnancy is the mpitaiza (traditional healer), who intervenes to lift curses or provide protection against acts of witchcraft. The women who had lost several fetuses often turn to this type of healer.

The women considered these different types of care to be complementary and not mutually exclusive. The objective of these types of care is to ensure the health of the mother and child, the healthy development of the fetus and an uncomplicated delivery. A delivery was perceived as “complicated” when a healthcare professional was required to remove the fetus, usually by cesarean section. The women feared undergoing this operation because they believed that it devalued them and imposed significant financial costs on the family.

### Determinants of antenatal care use

A multinomial regression showed that during the second trimester, having up to the primary school level improved the chance of pregnant women attending one or more ANC visits. The effect of this factor no longer seems to be significant in the second trimester. This was the only significant variable in the second trimester subset group. In contrast, in the third trimester of pregnancy, the women with multigravidity and those having occupation were less likely to attend 1–3 ANC visits and to consistently have 4 or more ANC visits (Table 5).

**Table 5:** Multinomial regression model that analyzes the determinants of antenatal care practices (*n* = 245)

**Variable/Category**	**Second trimester (*n* = 118)**				**Third trimester (*n* = 127)**			
	**0 VS 1 to 3 ANC**		**0 VS 4 ANC or more**		**0 VS 1 to 3 ANC**		**0 VS 4 ANC or more**	
	**RRR**	**CI95%**	**RRR**	**CI95%**	**RRR**	**CI95%**	**RRR**	**CI95%**
Type of Residence								
Urban (ref)	1		1		1		1	
Rural	2.11	[0.59–7.60]	1.01	[0.13–7.90]	0.27	[0.00–10.48]	0.09	[0.00–3.79]
Origin region								
Other (ref)	1		1		1		1	
Vakinankaratra	0.51	[0.06–4.18]	0.23	[0.02–2.57]	10.79**	[2.21–52.61]	12.12*	[1.54–95.51]
Occupation								
No (ref)	1		1		1		1	
Yes	0.35	[0.07–1.81]	0.21	[0.03–1.43]	7.51e-06***	[0.00–0.00]	4.58e-06***	[0.00–0.00]
Family income								
< = $1.90 USD per day (ref)	1		1		1		1	
> $1.90 USD	1.06	[0.36–3.11]	2.32	[0.35–15.48]	0.56	[0.09–3.51]	1.00	[0.14–7.09]
School level								
None or Primary school (ref)	1		1		1		1	
Over primary school	4.09*	[1.21–13.82]	6.61*	[1.28–34.06]	1.59	[0.14–18.09]	1.42	[0.12–17.68]
Family size								
0 to 3 members (ref)	1		1		1		1	
Over 3 members	0.83	[0.31–2.22]	0.22	[0.03–1.71]	0.22	[0.01–3.54]	0.10	[0.00–1.91]
Distance to PHF								
< = 5 km (ref)	1		1		1		1	
Over 5 km	0.43	[0.14–1.32]	1.03	[0.16–6.66]	0.26	[0.03–2.42]	1.17	[0.01–2.15]
Age (years)								
< = 24 (ref)	1		1		1		1	
25 or more	1.47	[0.47–4.57]	4.70	[0.48–46.02]	0.20	[0.02–2.33]	0.73	[0.06–9.14]
Gravidity								
Primigravida (ref)	1		1		1		1	
Multigravida	1.10	[0.22–5.51]	1.25	[0.10–14.96]	**5.48**^e−06^***	[0.00–0.00]	7.75e-06***	[0.00–0.00]
Preference for care								
Other^b^ (ref)	1		1		1		1	
Modern health system only	1.27	[0.49–3.28]	1.35	[0.26–7.21]	1.22	[0.06–23.20]	1.36	[0.06–28.80]

^***^p < 0.001, **p < 0.01, *p < 0.05

^a^*RRR* Relative Risk Ratio, ^b^ “Other” includes traditional only or both modern and traditional healthcare

Regarding ANC practices and from the qualitative analysis we identified two consistent factors which could impact healthcare seeking: the time of the first natal visit and appreciation of the ANC content.

#### Time of the first antenatal care visit

In the qualitative interviews, the women reported that they only began to use ANC when their pregnancies had progressed and their bellies became large. In fact, it is considered inappropriate to talk about or refer to a pregnancy before it is visible.

Other factors that influenced the start and frequency of antenatal visits were the lack of financial resources or the lack of time due to daily tasks. Therefore, for the pregnant women who already had children, their ANC began between the fourth and ninth months of pregnancy, specifically, only when the pregnancy was visible. Finally, some women chose to delay consultation at the healthcare center to reduce the number of iron tablets that they would have to take, as they reported that they found the tablets difficult to digest, despite the tablets being mandatory from the healthcare workers’ point of view.

#### Content and appreciation of antenatal care visits

Antenatal consultations often take place within a public or private institution. The women described antenatal visits as check-ups on the baby’s and mother’s health status and on monitoring of the fetus’s development by measuring the belly, checking the cervix, recording the mother’s weight and listening to the baby’s heartbeat. In some cases, the women described the doctor examining their eyes and tongue and identifying possible varicose veins. Preventive measures (e.g., deworming, vaccinations and iron tablets) were part of the care received during ANC. However, few women cited blood tests, screening tests (for STIs and HIV) and ultrasounds as part of the care provided during antenatal visits.

The women were generally positive about ANC visits. They especially appreciated the welcoming environment, the quality of care and the information sessions, which they considered to be useful for managing their pregnancy well. A few women had less favorable opinions of ANC visits when they were cared for by trainees: trainees were said to be tactless and to not provide any advice. As noted above, the women also appreciated receiving a booklet that listed the ANC visits made. This reassured them that they would be cared for in a healthcare facility in case of complications during childbirth.

### Utilization of a skilled birth attendant at delivery

**Fig. 1:**
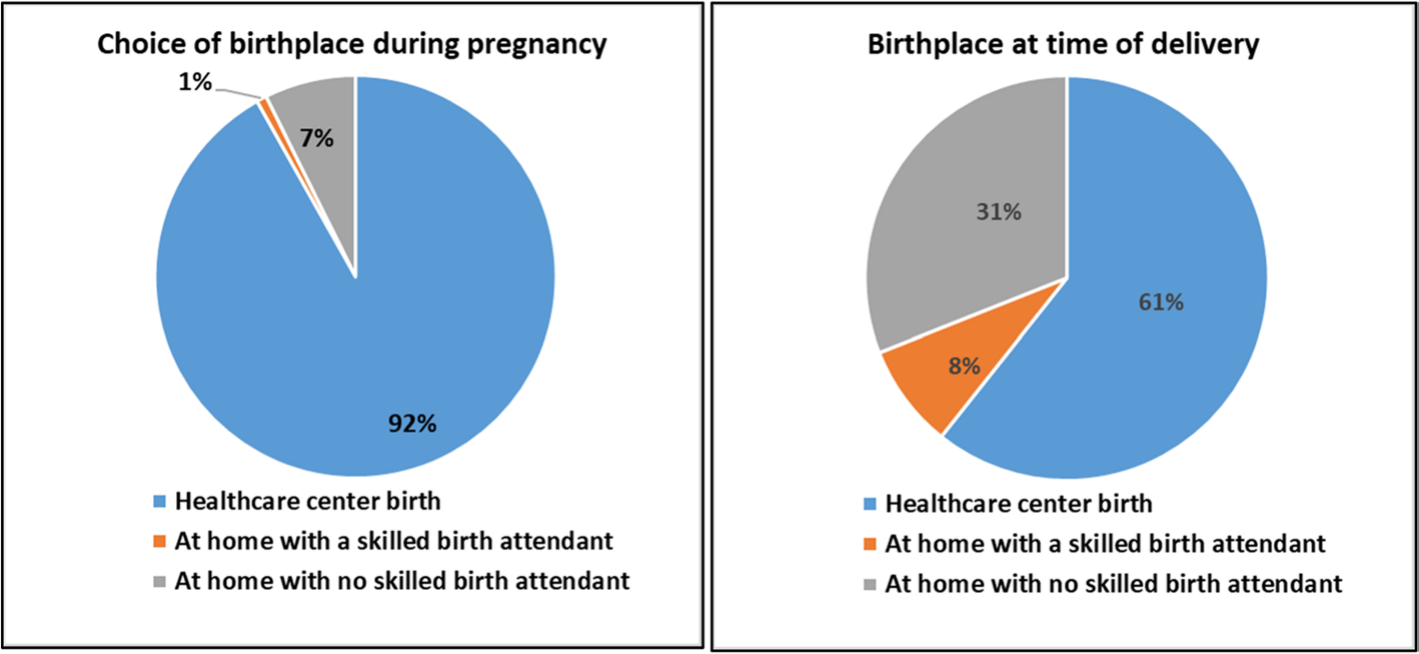
Desired and effective Birthplace

During pregnancy, 92% of the surveyed women expressed a desire to give birth at an HF. However, only 61% of the deliveries took place at an HF. The rest of the deliveries occurred at home, and among them, only 8.3% were assisted by an SBA (Fig. 1).

The regression analysis (Table 6) showed that the following 4 factors influenced the use of SBAs at delivery: the school level; the frequency of ANC; the origin region; and the preference for care (modern or traditional care). Therefore, the women who had at least 4 ANC follow-ups were more likely to be assisted by an SBA during delivery (AOR = 3.1; 95% CI [1.0–9.6]) than the women with fewer follow-ups. Similarly, the women with up to secondary educational levels and the women who used only biomedical healthcare services were significantly more likely to have an SBA at delivery (*p* < 0.001). The women living in rural areas were more likely to be assisted by an SBA at delivery (AOR = 2.4; 95% CI [1.0–5.3]) than the women living in urban areas. However, the odds of having SBA assistance during childbirth were lower for the women from the Vakinankaratra region (AOR = 0.1; 95% CI [0.0–0.8]) than for the women from other regions.

**Table 6:** Binary logistic regression analysis of the determinants of delivery at an HF (*n* = 229)

**Variables**	**AOR**	***CI95 min***	***CI95 max***
**ANC visits**			
*0 (ref)*	1		
*1 to 3*	1.72	0.70	4.25
*4 or more*	3.14*	1.03	9.57
**Type of residency**			
*Urban (ref)*	1		
*Rural*	2.35*	1.04	5.30
**Region**			
*Other region (ref)*	1		
*Vakinankaratra*	0.13*	0.02	0.78
**Occupation**			
*No (ref)*	1		
*Yes*	0.53	0.20	1.43
**Family income***(per day)*			
< = *$1.90 USD (ref)*	1		
> *$1.90 USD*	0.64	0.30	1.38
**School level**			
*None or Primary school (ref)*	1		
*Over primary*	5.87***	2.55	13.51
**Family size**			
*0 to 3 members (ref)*	1		
*Over 3 members*	0.94	0.44	1.44
**Distance to PHF**			
< = *5 km (ref)*	1		
*Over 5 km*	0.43	0.18	1.02
**Woman’s age***(years)*			
< = *24 (ref)*	1		
*25 or more*	0.77	0.32	1.83
**Gravidity**			
*Primigravida (ref)*	1		
*Multigravida*	0.86	0.30	2.43
**Preference for care**			
*Other*^a^	1		
*Healthcare system only*	3.59***	1.81	7.14

^***^p < 0.001, **p < 0.01, *p < 0.05

^a^ “Other” includes “only traditional” or “both modern and traditional healthcare”

Herein, the contextual factors around childbirth care practices are developed.

### Reasons for giving birth at healthcare facilities

The qualitative interviews confirmed that most of the women preferred to deliver at HFs. The reasons why they wanted to give birth at an HF whenever possible varied. Despite the expenses involved in an HF delivery, the women considered this option to be the best choice for themselves and their babies due to access to medical care. Delivery at an HF also facilitates the procedure of obtaining the baby’s birth certificate. For some mothers, HF delivery appeared to be the safest way to prevent and manage obstetric complications. The women who had already lost a child at birth expressed a clear preference for delivery at an HF.

A welcoming environment, the quality of services (follow-up during pregnancy) and a good relationship with caregivers also motivated the women to prefer to give birth at HFs. In addition, the interviews with caregivers (traditional healers and doctors) revealed that some *reninjaza* encouraged the pregnant women who they treated to deliver at an HF.

### Reasons for giving birth at home

The respondents’ reasons for home delivery were the inability to go to the healthcare center or hospital due to financial problems, the distance and travel time to HFs, and, in particular, the circumstances of the delivery that prevented them from going to an HF, including the absence of relatives or neighbors to accompany them to the HF, the suddenness of the birth, and the time of day of birth (most HFs are closed at night due to personnel constraints). In these cases, the family called a caregiver (midwife, *reninjaza* or female relative) to assist with the delivery. The husband/partner and close family members (e.g., mother, mother-in-law or sister) were the main people who decided at the time of delivery whether to take the woman to an HF or whether to call a reninjaza or a female relative to help her give birth. Therefore, the family plays a primary role at the time of childbirth.

Some women and their families had negative perceptions of HFs, especially at the hospital level, due to negative past experiences. Fear of the cesarean section operation and its implications (such as the costs, impact on the woman’s health and her ability to regain her strength) were mentioned.

Finally, some women chose to give birth at home with a *reninjaza* from their family network. In such cases, the neighborhood was called on to support and help the woman until the *reninjaza* arrived. Most of the time, the *reninjaza* arrived after the delivery and only managed the placenta, gave care to the newborn and cut the umbilical cord. Some women affirmed that they chose a *reninjaza* because of a strong interpersonal relationship. In most cases, the *reninjaza* was part of the family or lived in the same fokontany. Consequently, the women trusted in their services because of their familiarity, affectionate relationships or family links.

## Discussion

The results of this study showed that in the Vakinankaratra region, seeking maternal care from qualified medical staff is largely accepted and practiced in both rural and urban areas. The study eligibility criteria considered only pregnant women in the second or more trimester of pregnancy. In the same way, having conducted the second data collection a few months after childbirth allowed us to better investigate the practices and mainly reduced recall bias. Moreover, sampling some respondents for in-depth interviews from the women included in the quantitative analysis established a qualitative approach that ensured the cross-checking of information and provided better structured results. Finally, the consideration of parents and caregivers in the qualitative interview brought more elements of understanding through an in-depth analysis of the factors of maternal healthcare seeking.

### ANC practice and influencing factors

The relatively high number of ANC visits in the second and third trimesters of pregnancy potentially increased the possibility of having a good understanding of the medical actions performed during the visit and indicated a trend toward the adoption of this type of healthcare seeking. The quality of care provided by healthcare workers that was emphasized during the survey included the following standard elements: a welcoming environment; an explanation of the care; the provision of advice; and a nonjudgmental attitude in encouraging the use of ANC. In fact, inefficient communication by and a lack of information from health personnel during ANC have been reported to be barriers to maternal healthcare visits [25, 43–45]. So, Even if women would have initiated the ANC visits, these elements influenced the women’s decisions about completing visits and could even impact on giving birth at HFs.

If we look at the frequency of ANC in light of the new WHO recommendations, the data collected do not allow us to observe the number of ANC visits attended at each stage of pregnancy or the number of contacts with healthcare workers. However, the analyses of the determinants of ANC practice and other possible recourses allow us to understand the women’s perceptions of ANC and the constraints that they face to thus discuss the possibility of attending the 8 recommended visits.

Having completed primary school positively influenced the use of ANC in the second trimester but less so in the third trimester of pregnancy. Here, the assurance and comfort provided by the knowledge of women’s health status and that of their baby through the examinations conducted during ANC appointments could play a role. Indeed, the information provided early on in the first consultation(s), when it is reassuring, can lead educated women and their relatives to follow up on their pregnancy without feeling compelled to complete all the required ANC visits and to consider giving birth in the absence of assistance from qualified medical personnel. This situation is emphasized in the qualitative interviews and echoes similar situations reported in other studies [8].

The level of education has a different effect, as the women with a higher educational level could have potentially initiated ANC earlier, as recommended, but not completed it [46]. In the third trimester, almost all of the women used ANC regardless of their level of education [43–46].

Being from Vakinankaratra also affects the frequency of visits of women in the third trimester. In this region, almost all women mentioned the need for ANC but only attended ANC visits when the pregnancy was sufficiently advanced.

To the contrary, being economically active and having already had children negatively influence the practice of ANC in the third trimester. The qualitative interviews show that the activities to which women are assigned limit the number of ANC visits that they can attend. Similarly, among the multiparous women, most of whom have had no incidents of previous or current pregnancies, the frequency of ANC decreases in the third trimester, especially if they have to deal with their occupation and other constraints. The requirement for women to remain active and carry out daily and domestic activities until childbirth has been identified in another region of Madagascar [8]. This study showed that women are recognized and valued if they assume family and domestic tasks and income-generating and subsistence activities, even during pregnancy. In fact, in a low-income country such as Madagascar, active women, even those who are pregnant or are at an advanced age, are often observed. The only time women are prohibited from performing their daily activities is the postpartum period (mifana): some weeks/months following the birth of a child [8, 11].

The qualitative interviews also revealed that the women who had attended more than 4 ANCs were those who had already had problems at the time of previous deliveries (complications or stillbirth) and that they wanted to take the least risk for the current pregnancy. Indeed, the fear of complications is often reported as a reason for attending ANC visits [43, 44, 46, 47].

Women preferring modern care and women who completed 4 ANC visits or more were potential factors that increased the likelihood of delivery at HFs. This finding is in line with studies conducted in Ethiopia [24, 48, 49]. In fact, the women received advice and necessary information during these visits, which created a trust-based relationship between the caregivers and the pregnant women. Our study emphasizes that only 1 to 3 ANCs are insufficient to drive the seeking of SBAs at delivery when at least 4 ANC visits are needed. This is consistent with respect to the continuum of care from the prenatal period to the time of delivery [50].

Regarding the WHO current recommendations, which emphasize a minimum of 8 healthcare contacts or ANC visits during the pregnancy period, our study did not allow us to carry out a consistent analysis regarding the number of contact visits, as the MH operational implementation of these recommendations took place in Madagascar only 3 years after this study’s data collection. Nevertheless, the national recommendation adopted by the local MH is to have at least 8 contacts with qualified healthcare personnel, including at least 4 ANC visits. Thus, the potential recommendations of our study drawn from the results of our analyses are to some extent compliant with the new directives of the local MoPH and are therefore adapted to the reality of the country.

### Determinants of the use of an SBA at delivery

Women from Vakinankaratra are less likely to give birth with an SBA than women from other regions. Women from other regions have less opportunity to give birth with a *reninjaza* because they do not live in their home community. Using a *reninjaza* involves a close, long-term relationship. They are therefore more likely to deliver in a healthcare facility.

Women’s school level seems to consistently be an influencing factor in different sub-saharian countries for seeking SBAs [22, 46]. In fact, counseling and awareness messages, which often have medical terms and content, can be quite accessible for women with a primary school educational level. However, even when pregnant women exhibit danger signs related to pregnancy complications, it may be difficult for them to make wise decisions about their own health. Then, at the time of delivery, the decision of where the delivery will take place is mainly made by the woman’s partner or her relatives. Indeed, an entourage, especially partners, plays a crucial role in maternal healthcare seeking [45, 51–54]. Thus, it is necessary to strengthen women’s autonomy and to include other members of the family in incentive programs for maternal healthcare. It is also necessary to ensure that childbirth in healthcare facilities, including cesarean sections, is effectively free [27, 28].

Generally, women living in rural areas seek fewer maternal healthcare services than women living in urban areas [25, 44, 46, 55, 56]. In Vakinankaratra, the women living in rural areas were more likely to seek SBAs for delivery than the women living in urban areas. This finding, which seems unusual, could be linked to the close geographical proximity between the two sites to one another (-20 km), which would have minimized the impact of the differences in the geographical access of women to healthcare centers. In addition, nearly 83% of the women who participated in the study lived within 5 km or less of an HF, and it is known that reducing the travel time to an HF is crucial in motivating and encouraging women and their families to give birth at HFs [25, 44, 46, 55, 56].

We also found that some *reninjaza* encouraged women to seek ANC and to deliver at HFs when they predicted childbirth to be complicated. Therefore, the collaboration between traditional healers and HFs should be reinforced and sustained for better health message communication. An analysis of the relationship between the medical staff and *reninjaza* in other regions of Madagascar (Alaotra Mangoro, Vatovavy Fitovinany, Menabe, and Antananarivo) underscored the possibility of an informal collaboration between these birth attendants when there was mutual recognition of one another’s unique and complementary skills [7].

Among the women who gave birth at home, the circumstances of the birth, including financial problems and travel to an HF, were emphasized through the qualitative study as the main barriers to delivery at HFs. The women began to save money only just before the delivery, and in most cases, the savings were largely insufficient to pay for medical care access. In fact, in Malagasy culture, only the event itself triggers action; therefore, in this context, no financial preparation is made for childbirth [57, 58]. Accordingly, implementing specific strategies for low-income households to support women and families to better plan childbirth (e.g., free-of-charge maternal services and the provision of necessary consumables) is very important to encourage delivery at HFs, which may, in turn, help to reduce maternal deaths.

## Conclusion

The use of maternal healthcare in the Vakinankaratra region is on track to be adopted by women. Women are aware about maternal health, and the majority of them use maternal healthcare services. Women with an educational level above primary school practice more ANC slightly earlier. At the same time, women seek TBA for many aspects of care that are not available at healthcare facilities. In addition, women have a positive perception of ANC and childbirth in a healthcare facility (except for those who have had negative experiences). However, a range of sociocultural and economic factors restrict their use of healthcare facilities for ANC or delivery. Thus, ANC is perceived as necessary by women from the moment that the pregnancy becomes apparent to ensure the good health of the mother and baby. However, women do not perceive the need to multiply ANC, especially since they have to maintain their domestic and subsistence activities until the birth of their child.

The factors that positively influenced the seeking of SBA services at childbirth were mothers’ formal educational level, a high frequency of ANC visits and a preference for modern care. The main barriers were the cost of travel to the healthcare facility (and the need for the woman to be accompanied) and the cost of care and equipment needed to deliver in a healthcare facility. Even if the delivery itself was free, the informal costs remained high. The lack of financial autonomy and decision making by women remains the main barrier to childbirth assisted by an SBA. Women’s relatives play a crucial role (in logistic support and decision making) at the time of delivery. Being unable to plan ahead for travel to the healthcare facility (due to social norms and financial limitations) is also a barrier.

Despite these limitations, the results confirm the multidimensional nature of the interventions needed to improve maternal healthcare seeking. For women, education and women’s empowerment seem to be basic and imperative. At the community level, the extension of sensitization concerning maternal health to other family members and to community representatives is necessary to make them aware of their roles related to maternal health and to involve them in taking measures for maternal healthcare seeking. In addition, the healthcare services offered must go beyond skills to include communication and respect. The use of multiple forms of care continues to be practiced and must be taken into account in strategies to better meet all of the healthcare needs of pregnant women and mothers. From this perspective, collaborations between traditional healers and biomedical caregivers should be supported and formalized. Further investigation must be carried out to better understand the interregional disparities in maternal care practices in Madagascar.

This study has some limitations. An analysis of the practice of the 8 prenatal contacts, which was recently recommended by the WHO, was not possible. We focused on formal ANC visits because the concept of contacts was not well understood and was not tracked within by the MH at the time of the study. This somewhat limits the scope of our recommendations. Regarding the qualitative study, we were not able to make observations at the time of the deliveries. This would have allowed us to observe the interactions and to better understand the issues surrounding the decision-making concerning the place of delivery and the people involved.

## Data Availability

The datasets used and analyzed during the current study are available from the corresponding author on reasonable request.

## Abbreviations

ANC: Antenatal care
AOR: Adjusted odds ratio
BIRDY: Bacterial infections and antibiotic resistant deases
CHW: Community health worker
CI: Confidence interval
DHS: Demographic Health survey
HF: Healthcare facility
HIV: Human Immunodeficiency Virus
IDIs: In-depth interviews
IEC: Information and Education Community
IRD: Institute of Research for Development
ITN: Insecticide treated net
JEAI: Jeunes équipes associées IRD
JSI: John Snow, Inc.
MICS: Multiple indicators cluster survey
MH: Ministry of health
NHMIS: National health management information system
PHF: Primary Healthcare Facility
RESOFEN: Healthcare seeking for pregnant women and children in Madagascar
RRR: Relative risk ratio
SARA: Service Availability and Readiness Assessment
SBA: Skilled birth attendant
SDG: Sustainable development goal
STI: Sexually transmissible infections
TBA: Traditional birth attendant
USAID: US Agency for International Development
WHO: World Health Organization
